# A systematic review of multimodal prehabilitation in breast cancer

**DOI:** 10.1007/s10549-022-06759-1

**Published:** 2022-10-21

**Authors:** Kellie Toohey, Maddison Hunter, Karen McKinnon, Tamara Casey, Murray Turner, Suzanne Taylor, Catherine Paterson

**Affiliations:** 1 Faculty of Health, University of Canberra, Bruce ACT, 2617 Australia; 2 Prehabilitation, Activity, Cancer, Exercise and Survivorship (PACES) Research Group, University of Canberra, Bruce ACT, Australia; 3 Australian Capital Territory Breast Care, Calvary Public Hospital, Bruce ACT, Australia; 4 Robert Gordon University, Aberdeen, AB10 7QB Scotland

**Keywords:** Exercise, Nutrition, Nursing, Psychology, Wellness, Cancer care

## Abstract

**Purpose:**

Breast cancer is the most prevalent malignancy in women. Prehabilitation may offer improvements in physical and psychological wellbeing among participants prior to treatment. This systematic review aimed to determine the efficacy of prehabilitation in participants diagnosed with breast cancer.

**Methods:**

A systematic review was performed according to Preferred Reporting Items for Systematic Reviews and Meta-Analyses (PRISMA) Guidelines. Studies exploring the impact of prehabilitation in participants with breast cancer were included. Studies were assessed independently according to pre-eligibility criteria, with data extraction and methodological quality assessed in parallel.

**Results:**

3184 records were identified according to our search criteria, and 14 articles were included. Articles comprised of quantitative randomised controlled trials (*n* = 7), quantitative non-randomised studies (*n* = 5), a qualitative study (*n* = 1), and a mixed-method study (*n* = 1). The majority of selected studies completed exercise programs (*n* = 4) or had exercise components (*n* = 2), with two focusing on upper-limb exercise. Five articles reported complementary and alternative therapies (*n* = 5). Two articles reported smoking cessation (*n* = 2), with a single study reporting multi-modal prehabilitation (*n* = 1). Mostly, prehabilitation improved outcomes including physical function, quality of life, and psychosocial variables (*P* < 0.05). The qualitative data identified preferences for multimodal prehabilitation, compared to unimodal with  an interest in receiving support for longer.

**Conclusions:**

Prehabilitation for patients with breast cancer is an emerging research area that appears to improve outcomes, however, ensuring that adequate intervention timeframes, follow-up, and population groups should be considered for future investigations.

**Implications for Cancer Survivors:**

The implementation of prehabilitation interventions for individuals diagnosed with breast cancer should be utilised by multidisciplinary teams to provide holistic care to patients as it has the potential to improve outcomes across the cancer care trajectory.

**Supplementary Information:**

The online version contains supplementary material available at 10.1007/s10549-022-06759-1.

## Introduction

Cancer incidences and mortality rates continue to grow across the world [1]. Evidence identifies that 2.1 million new breast cancer (BC) diagnoses are made annually accounting for one in four cancers among females. Breast cancer is the most commonly diagnosed cancer in females and the leading cause of death [1] globally. The highest BC incidence rates include Australia with a 5-year relative survival rate (2013–2017) of 92%. In 2021, it was estimated that 20,030 new cases of BC will be diagnosed in Australia [2]. Due to improved survival rates over the past two decades (1988–1992 and 2013–2017 improved 76–92% [2], respectively), many individuals diagnosed with BC are now living with the long-term effects of the diagnosis including debilitating treatment effects with a number of unmet supportive care needs [3, 4].

Compared to individuals without cancer, BC survivors are at an increased risk of developing anxiety and depression, fear of recurrence, sexual dysfunction, and relationship issues [5, 6]. It has also been reported that the transition from being a BC patient to a survivor can be associated with increased physical and psychological challenges associated with unmet needs [5]. Programs to support the complex needs of individuals with BC are currently ad hoc and urgently need evaluation and optimisation to support this ever-growing population from diagnosis through to survivorship [4]. Supportive care considers and addresses the physical, emotional, social, spiritual, and informational needs of people diagnosed with cancer throughout the disease trajectory [7]. The delivery of supportive care for people diagnosed with BC continues to be suboptimal [8]. People and their families are required to seek out multiple interventions which focus on improving quality of life (QOL) and rehabilitation which has significant financial impact [9–12].

Strong evidence suggests that lack of physical activity (PA) is associated with an increased risk of BC and poorer outcomes for those diagnosed [13–15]. Physical inactivity and unhealthy behaviours contribute to the disease burden for individuals with BC and the Australian Burden of Disease study indicated that physical inactivity contributed 6.4% of the burden [16, 17]. There is an inverse relationship between PA (a modifiable risk factor for BC) and all-cause mortality, BC-related death, and BC events [18]. Appropriate PA interventions may be important for people diagnosed with BC and their families to reduce mortality and BC recurrence [19]. The Clinical Oncology Society of Australia’s (COSA) position statement recommends that people going through cancer treatment participate in 150 min of moderate intensity PA, or 75 min of vigorous activity per week, along with two resistance-based sessions per week [20], which is the same recommendation published by the World Health Organisation (WHO) for healthy individuals [21]. COSA also recommends that optimal care is achieved by matching services and resources to the individual persons’ requirements, which are then easily accessible and integrated with the multidisciplinary team (MDT) [22]. Even though PA guidelines and recommendations exist for participants with BC, they do not address the complexities of their overall supportive care needs including prior to surgery in the prehabilitation period. Prehabilitation is acknowledged to be the presurgical period where effective programs could be used to optimise the physical and emotional status of the patient before the stress of their operation and could be key to addressing participant’s individual needs [23].

The model of survivorship care published by COSA in 2016 [24] suggested that to achieve optimal care, services, and resources need to be carefully matched to the specific persons needs and concerns, and it needs to be accessible to the person and integrated across the MDT [24]. Currently gaps remain and this level of care is not accessible to many individuals with BC across models of care and in current survivorship pathways [25–27]. Medical follow-up often overlooks a person’s psychosocial issues and important referral needs, leading to suboptimal supportive care [28, 29]. This will then be further impacted by the lack of guidance for the person as their needs have not been highlighted and addressed. Prehabilitation could be used as a time to address the needs of individuals with BC by using the most appropriate tools to identify necessary multimodal referral pathways [30–32]. The prehabilitation phase of treatment could be utilised to identify patient concerns, establish referral pathways, and set review timeframes to ensure a smooth cancer treatment journey [24].

Multimodal interventions which include MDT care are inconsistent and underutilised for individuals with BC [3]. The inclusion of multimodal, MDT programs recognises the need for supportive care programs which includes a holistic approach to peoples’ wellness across their personal BC recovery [33]. Therefore, the aim of this systematic review was to determine what supportive care prehabilitation programs exist to assist those diagnosed with BC and what outcomes were reported.

## Methods

This systematic review has been reported in accordance with the Preferred Reporting Items for Systematic Reviews and Meta-Analyses (PRISMA) guidelines [34] and was registered in PROSPERO International Register of Systematic Reviews (CRD42021259463), available from https://www.crd.york.ac.uk/prospero/display_record.php?RecordID=259463.

### Literature search

An electronic database search using combinations of MeSH and free-text words for “breast cancer “ and “prehabilitation” were undertaken by an experienced health information specialist (MT) using the following databases: CINAHL and Medline on EBSCOhost platform, Cochran Library (DSR and CENTRAL), Scopus, and Web of Science Core Collection and were conducted on 1 July, 2021 and updated on the 21 March, 2022. The Participant, Intervention, Comparator, and Outcome (PICO) framework [35] was used to define the inclusion criteria.

#### Participants

Participants were included if they were > 18 years, had a diagnosis of primary BC, and participated in a prehabilitation program of any kind prior to treatment.

#### Intervention

Clinical trials, cohort studies (retrospective and prospective), and case control studies that explored prehabilitation programs for participants with BC prior to invasive surgery. Studies developing, validating, updating, evaluating, or comparing prediction models of BC were eligible. Only studies published in English were included. The following articles were excluded: Review articles and studies published in languages other than English, protocols, conference abstracts, and clinical trial registrations were also excluded. Eligible studies were characterised into subgroups based on the type of prehabilitation program.

#### Comparators

Studies that compared prehabilitation to usual care or no prehabilitation or another intervention were included.

#### Outcomes

Studies that evaluated the feasibility and/or the effectiveness of the prehabilitation intervention(s) on health-related outcomes (e.g. quality of life, physical outcomes) were eligible.

### Data extraction and management

Search results that were identified during the electronic database search were transferred to Covidence, a systematic review software (Version 2579, Melbourne, Australia) and duplicate titles removed. Title and abstracts were resolved by five authors, and conflicts were by discussion. Articles then considered potentially eligible were moved to the full-text screening. Full-text articles were independently assessed by a minimum of two reviewers. All authors extracted the following outcomes compared to baseline from each study into table format; population, outcomes, physical function assessments, clinical assessments, patient-reported outcome measurement, and findings. All data extraction was quality checked by a second reviewer.

### Assessment of study quality

Methodological quality assessment of the included studies was completed using the Mixed Methods Appraisal Tool (MMAT 2018) [36]. The quality assessment was carried out by all authors during the data extraction phase, and a second author then quality checked assessments on all articles, discussing any disagreements.

## Results

### Study selection

**Fig. 1:**
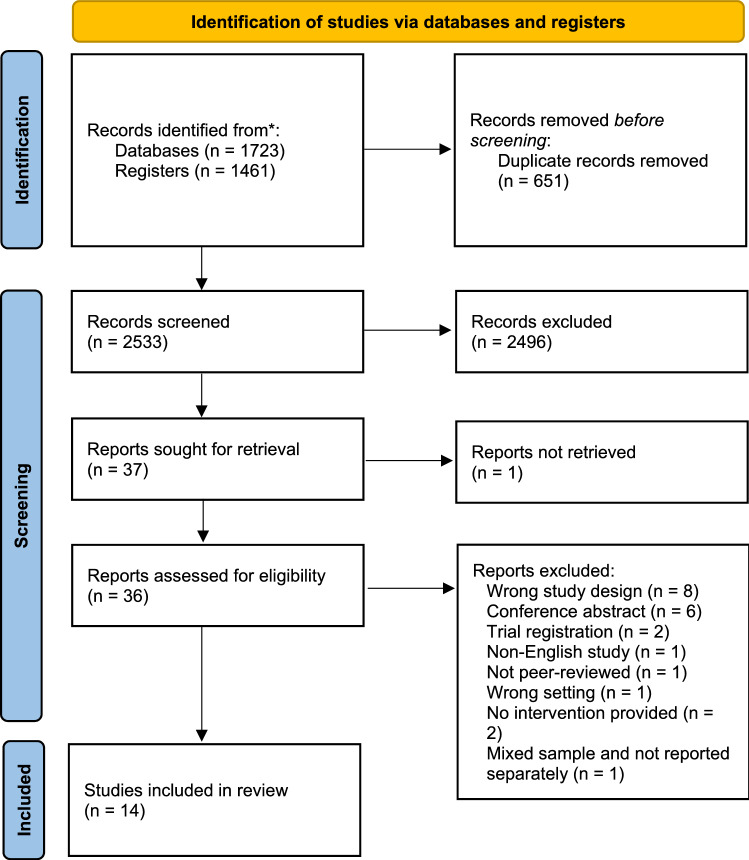
Search strategy and article selection process according to the Preferred Reporting Items for Systematic Reviews and Meta-Analyses (PRISMA) Guidelines [34]

The literature search of electronic databases and registers (Fig. 1) identified 3184, with secondary searches of the retained full-text articles reference lists revealing no further articles. Following the removal of duplicates (*n* = 651) and irrelevant studies based on the application of the pre-screening eligibility criteria, 36 full-text articles were assessed. One full text was not able to be retrieved. A total of 22 articles were excluded with reasons and 14 articles met the inclusion criteria [37–50]. Of note, two publications (Thomsen et al. [46] and Thomsen et al. [47]) reported different data from the same study. The articles comprised quantitative randomised controlled trials (*n* = 7) [39–41, 44, 45, 47, 48, 50], quantitative non-randomised studies (*n* = 5) [37, 42, 43, 48, 49], a qualitative study (*n* = 1) [46], and a mixed method study (*n* = 1) [38].

### Study characteristics

The characteristics of the included studies are described in Table 1. A total of 1,568 participants (quantitative participants *n* = 1,529; qualitative participants *n* = 11; mixed-methods participants *n* = 28) were included. Noteworthy, there was only one male participant included across all studies, and it was unclear whether this participant completed the intervention or was lost to follow-up. The included participants were heterogeneous in clinical characteristics including cancer stage, type of BC, and type of treatment, which included surgery and surgery type, chemotherapy, radiotherapy, and hormonal therapy, which deemed the completion of a meta-analysis inappropriate. For the quantitative studies, the samples ranged from 41 to 400, with an average age range 42–63 years. The sample size for the qualitative study was 11 and the age range was 40–72 years, with the mixed-methods study included 28 participants, with a mean age of 54 ± 10.98. The included studies were carried out in United States of America [37, 40, 41, 43], Canada [38], Sweden [39], Japan [42, 44, 45], Denmark [46, 47], China [48], United Kingdom [49], and Romania [50].

**Table 1:** Overview of included studies

Author/Year	Purpose	Sample size/mean age (SD, years), gender	Participants (Cancer type, cancer stage, treatment)	Response rate (reasons for declining); attrition/adherence	Design	Time points	Data collection tools
Baima et al. 2017 USA [37]	Explore the feasibility of an independent home shoulder exercise program to improve ipsilateral shoulder pain and abduction ROM after BC surgery	**Sample size**: *n* = 60 (Group 1: *n* = 36; Group 2: *n* = 24)	**Type:** Not reported	**Response rate:** *n* = 64 recruited, *n* = 60 consented; *n* = 45 completed study (accessibility of study staff at follow-up appointments, delayed surgical treatment due to prolonged CT, disease worsening)	Prospective, cohort observational study	T1: 1–4 weeks prior to surgery	Subjective data collection: Pain scale (0–10)
		**Age:** 35–81 years (median not reported)	**Stage**: Not reported	**Attrition/adherence:** 76% chose to exercise; *n* = 29 (85%) performed exercises 3 ≥ per week		T2: 3 months post-surgery	Objective data collection: ROM (0 to 180°); chart documentation of postoperative seroma formation
		**Gender:** Female: *n* = 59	**Treatment:** Surgery (not specified)				
		Male: *n* = 1					
Brahmbhatt et al. 2020 Canada [38]	Determine the feasibility and acceptability of an individualised, home-based prehabilitation intervention prior to BC surgery	**Sample size:** *n* = 28	**Type:** Not reported	**Response rate:** *n* = 45 approached, *n* = 28 consented (62%) ×××*n* = 28 consented (travel/distance (*n* = 3), extremely anxious (*n* = 2), travelling (*n* = 1), already active (*n* = 1), not interested (*n* = 5), no reason provided (*n* = 5) ×××*n* = 22 completed baseline: could not contact (*n* = 4), withdrew consent (*n* = 1), change in treatment plan (*n* = 1) ×××*n* = 18 completed T1 (lost to follow-up (*n* = 2), travelling (*n* = 1), not interested (*n* = 1) ×××*n* = 15 completed T2: lost to follow-up (*n* = 2), family commitments (*n* = 1) ×××*n* = 14 completed T3: illness (*n* = 1)	Prospective, single-arm, feasibility study with an emergent, embedded mixed-methods design	Baseline: lasted 30 ± 16.59 days	Subjective data collection Quantitative: DASH; FACT-F; SF-36, GLTEQ-LSI, WHODAS 2.0, BPI Qualitative: Semi-structured interviews with open ended questions
		**Age:** Mean age 54 ± 10.98 years	**Stage**: Not reported	**Attrition/adherence:** Overall attrition rate was 36% 17 participants submitted exercise logs Adherence to: minimum aerobic exercise prescription: 142.22 ± 82.66%; minimum resistance training prescription: 114.44 ± 38.26%; *n* = 13 (76%) participants completed > 70% prescribed exercise; *n* = 2 participants completed < 70% prescribed exercise in some sessions; *n* = 2 participants completed < 70% of prescribed exercise		T1: 1 week prior to surgery	Objective data collection: 6MWT; upper-extremity strength (handgrip dynamometry); manual muscle testing (digital handheld dynamometer); WC; BMI; lean body mass; BF%; fat mass; clinical disease related data
		**Gender:** Female: *n* = 28	**Treatments:** Unilateral lumpectomy with SLNB: *n* = 12 (54.55%); Unilateral mastectomy with SLNB: *n* = 2 (9.09%); Unilateral mastectomy with ALND: *n* = 1 (4.55%); Bilateral mastectomy with SLNB: *n* = 4 (18.18%); Bilateral mastectomy with SLNB and insertion of tissue expanders: *n* = 1 (4.55%), Bilateral mastectomy with immediate autologous reconstruction: *n* = 1 (4.55%)			T2: 6 weeks post-surgery	
						T3: 12 weeks post-surgery	
Heiman et al. 2021 Sweden [39]	Evaluate whether an intervention consisting of recommended physical activity before and after surgery improved physical recovery at 4 weeks after BC surgery	**Sample size:** *n* = 400 (I: *n* = 200; C: *n* = 200)	**Type:** Not reported	**Response rate:** *n* = 997 approached (*n* = 288 met exclusion criteria, *n* = 309 declined to participate), *n* = 400 consented, *n* = 370 completed primary endpoint, *n* = 368 analysed (I: *n* = 179, C: *n* = 189) Withdrew consent at preop phase: I: *n* = 17, C: *n* = 3 Withdrew consent at postop phase: I: *n* = 3, C: *n* = 7 Withdrew consent at analysis phase: I: *n* = 1, C: *n* = 1 **Attrition/adherence:** *n* = 318 (85.9%) (I: *n* = 149 (82.8%), C: *n* = 169 (88.9%)) returned baseline and 4-week questionnaires *n* = 151 (83.9%) intervention group returned physical activity diary	Randomised, controlled, multicentre open-label trial	Baseline: preoperative	Subjective data collection: SGPALS (AUDIT-C)
		**Age:** I: 30–84 years; median age 61 (IQR 52–68) years C: 38–89 years; median age 63 (IQR 54–71) years	**Stage:** Not reported			T1: 4 weeks postoperative	Objective data collection: ASA physical status grade, use of drainage, antibiotic, and thromboembolic prophylaxis, duration of hospital stay, complications, reoperations, and readmissions were retrieved from medical records
		**Gender:** Female *n* = 400	**Treatments:** Breast-conserving surgery: I: *n* = 147; C: *n* = 154; Bilateral surgery: I: *n* = 3; C: *n* = 7 Mastectomy: I: *n* = 36; C: *n* = 43; Bilateral surgery: I: *n* = 6; C: *n* = 4; Direct reconstruction: I: *n* = 2; C: *n* = 4 Missing: I: *n* = 17; C: *n* = 3			T2: 12 months postoperative	
Knoerl et al. 2022 USA [40]	Explore the impact of exercise and mind–body prehabilitation interventions on changes in quality of life and cancer treatment–related symptoms in women with newly diagnosed BC	**Sample size:** *n* = 49 (Exercise: *n* = 27; Mind–Body: *n* = 22)	**Type:** Not reported	**Response rate:** *n* = 49 consented, *n* = 47 completed T1, *n* = 46 completed T2, *n* = 35 completed T3	Randomised controlled trial	T1: at enrolment	Subjective data EORTC QLQ C30; HADS; PSS
		**Age:** Exercise: 53.3 ± 9.6. years Mind–Body: 53.4 ± 8.0 years	**Stage:** Stage 1: Exercise: *n* = 10; Mind–Body: *n* = 9 Stage 2: Exercise: *n* = 9; Mind–Body: *n* = 9 Stage 3: Exercise: *n* = 6; Mind–Body: *n* = 1 Unknown: Exercise: *n* = 1; Mind–Body: *n* = 1	**Attrition/adherence:** Participants in Exercise group increased physical activity by 203 min per week, participants in Mind–Body group completed 23 min *n* = 14 (66.7%) participants in Mind–body group returned diary; engaged with the intervention on 69% of pre-surgery days		T2: postintervention/immediately prior to surgery	Objective data 7-Day PAR
		**Gender:** Female *n* = 49	**Treatments:** Planning to undergo BC surgery			T3: 1-month post-surgery	
Larson et al 2000 USA [41]	Evaluate the feasibility and potential immunological benefit of a presurgical psychosocial intervention for BC patients	**Sample size:** *n* = 41 (I: *n* = 23; C: *n* = 18)	**Type:** Not reported	**Response rate:** *n* = 41 consented, *n* = 2 participants dropped out of control after T1	Randomised controlled trial	T1: within one week of diagnosis and prior to the intervention prior to surgery	Subjective data: CES-D, DES-IV, IES, LOT, SF-36
		**Age:** 29–80 years; mean 56 ± 13 years	**Stage:** Reported by *n* = 28 (68%) of participants: Stage 1: *n* = 17 (60.7%) Stage 2: *n* = 8 (28.6%) Stage 3: *n* = 2 (7.1%) Stage 4: *n* = 1 (3.6%)	**Attrition/adherence:** Not reported		T2: following the intervention but within 1–3 days	Objective data: NK cell activity and IFN-γ production
		**Gender:** Female: *n* = 41	**Treatments:** All were awaiting either surgery, or surgery plus RT or CT			T3: 1-week post-surgery	
Sato et al 2014 Japan [42]	Investigate the effectiveness of a perioperative education program for improving upper arm dysfunction in patients with BC	**Sample size:** *n* = 162 (I: *n* = 96, C: *n* = 66)	**Type:** Not reported	**Response rate:** *n* = 162 enrolled, *n* = 49. analysed: ALND: I: *n* = 39, C: *n* = 30 SLNB: I: *n* = 51, C: *n* = 29 T1: ×××I: *n* = 3 dropped out (*n* = 2 lost to follow-up, *n* = 1 changed hospital) ×××C: *n* = 5 dropped out (*n* = 2 lost to follow-up, *n* = 2 changed hospital, *n* = 1 lost interest) T2: ×××I: *n* = 3 dropped out (*n* = 1 lost to follow-up, *n* = 1 lost interest, *n* = 1 lack of time) ×××C: *n* = 2 dropped out (*n* = 1 lost to follow-up, *n* = 1 lack of time)	Controlled trial (allocated according to participant wishes)	Baseline: pre-surgery	Subjective data: SPOFIA; DASH
		**Age:** ALND: I: 52.9 ± 10.1 years C: 52.1 ± 12.9 years SLNB: I: 54.3 ± 10.6 years C: 53.7 ± 9.5 years	**Stage:** ALND: Stage 0: I: *n* = 0, C: *n* = 6.7% Stage 1: I: *n* = 7.7%, C: *n* = 20% Stage 2: I: *n* = 43.6%, C: *n* = 50% Stage 3: I: *n* = 41%, C: n = 23.3% Stage 4: I: *n* = 7.7%, C: *n* = 0 SLNB: Stage 0: I: *n* = 25.5%, C: *n* = 44.8% Stage 1: I: *n* = 54.9%, C: *n* = 48.3% Stage 2: I: *n* = 17.6%, C: *n* = 6.9% Stage 3: I: *n* = 2%, C: *n* = 0 Stage 4: I: *n* = 0, C: *n* = 0	**Attrition/adherence:** Not reported		T1: 1-week post-surgery	Objective data: Arm girth; shoulder ROM; Grip strength (dynamometer)
		**Gender:** Not specified	**Treatments:** Total Mastectomy: ALND: I: *n* = 56.4%, C: *n* = 36.7%; SLNB: I: *n* = 21.6%, C: *n* = 24.1% Partial Mastectomy: ALND: I: *n* = 43.6%, C: *n* = 63.3%; SLNB: I: *n* = 78.4%, C: *n* = 75.9% Adjuvant CT: ALND: I: *n* = 79.5%, C: *n* = 86.7%; SLNB: I: *n* = 11.8%, C: *n* = 24.1% Adjuvant RT: ALND: I: *n* = 69.2%, C: *n* = 83.3%; SLNB: I: *n* = 58.8%, C: *n* = 51.7% Adjuvant hormone therapy: ALND: I: *n* = 82.1%, C: *n* = 63.3%; SLNB: I: *n* = 80.4%, C: *n* = 75.9%			T2: 1-month post-surgery	
						T3: 3 months post-surgery	
Springer et al. 2010 USA [43]	Determine the extent and time course of upper-limb dysfunction in subjects seen pre-operatively and followed prospectively using a novel physical therapy surveillance model post-BC and treatment	**Sample size:** *n* = 94	**Type:** DCIS: *n* = 11 (11.70%) IDC: *n* = 44 (46.81%) DCIS and IDC: *n* = 31 (32.98%) Other: *n* = 8 (6.51%)	**Response rate:** *n* = 200 enrolled, *n* = 94 (47%) included in analysis	Prospective observational cohort study	T1: pre-surgery	Subjective data: ULDQ.
		**Age:** Mean age 53.39 ± 11.8	**Stage:** Stage 0: *n* = 11 Stage 1: *n* = 40 Stage 2: *n* = 30 Stage 3: *n* = 13	**Attrition/adherence:** Not reported		T2: 1-month post-surgery	Objective data: Shoulder ROM and strength; upper-limb volume and girth
		**Gender:** Female: *n* = 94	**Treatments:** BCT: *n* = 41 (43.62%) MRM: *n* = 50 (53.19%) Simple mastectomy: *n* = 3 (3.19%) ALND: *n* = 66 (70.21%) SLNB: *n* = 20 (21.28%) CT: *n* = 57 (60.64%) RT: *n* = 64 (68.9%) Hormone therapy: *n* = 67 (71.28%),			T3: 3–6 months post-surgery	
						T4: 12-months post-surgery	
Tamaki et al. 2017 Japan [44]	Compare the effects of aromatherapy on mood, quality of life, and physical symptoms in patients with BC	**Sample size:** n = 162 (I: *n* = 110; C: *n* = 52)	**Type:** DCIS: I: *n* = 10, C: *n* = 12 IDC: I: *n* = 78, C: *n* = 82 ILC: I: *n* = 10, C: *n* = 0 Others: I: *n* = 2, C: *n* = 6	**Response rate:** *n* = 249 approached, *n* = 162 consented	Pilot randomised controlled trial (2:1 randomisation)	Baseline: at time of hospitalisation	Subjective data: EORTC QLQ-C30
		**Age:** I: 52 ± 11.3 years C: 55 ± 13.5 years	**Stage:** Stage 0: I: *n* = 10%, C: *n* = 12% Stage 1: I: *n* = 50%, C: *n* = 47% Stage 2: I: *n* = 35%, C: *n* = 37% Stage 3: I: *n* = 5%, C: *n* = 4%	**Attrition/adherence:** I: *n* = 8 did not complete questionnaires, C: *n* = 1 did not complete questionnaires		T1: day of surgery	Objective data: Hypnotics, vital signs: blood pressure, heart rate, adverse events
		**Gender:** Not specified	**Treatments:** Mastectomy: I: *n* = 32%, C: *n* = 39%			T2: 1 day post-surgery	
Tanaka et al. 2021 Japan [45]	Assess the effects of Yokukansan on BC patients undergoing a practical or total mastectomy breast surgery	**Sample size:** *n* = 100 (I: *n* = 50, C: *n* = 50)	**Type:** Not reported	**Response rate:** *n* = 100 consented, *n* = 77 analysed (I: *n* = 35, C: *n* = 42) T1: ×××I: *n* = 3 excluded (*n* = 1 protocol violation, *n* = 2 refusal of study participation) T2: ×××I: *n* = 7 excluded (*n* = 7 positive sentinel lymph node) ×××C: *n* = 6 excluded (*n* = 5 positive sentinel lymph node, *n* = 1 inhaled steroid for treatment of bronchospasm T3: ×××I: *n* = 5 excluded (*n* = 5 incomplete answers to questionnaires) ×××C: *n* = 2 excluded (*n* = 2 incomplete answers to questionnaires)	Single-blind randomised controlled trial	T1: One day pre-surgery	Subjective data VAS; HADS; STAI; QoR-15
		**Age:** I: 49 ± 6.2 years C: 48 ± 5.7 years	**Stage:** Not reported	**Attrition/adherence:** Not reported		T2: immediately prior to surgery	Objective data sAA
		**Gender:** Female: *n* = 91	**Treatments:** Partial resection: I: *n* = 22, C: *n* = 21 Total mastectomy: I: *n* = 21, C: *n* = 27			T3: 1 day post-surgery	
Thomsen et al. 2009 Denmark [46]	Explore how women smokers with newly diagnosed BC experienced brief preoperative smoking cessation intervention in relation to BC surgery	**Sample size:** *n* = 11	**Type:** Not reported	**Response rate:** Not reported	Qualitative, descriptive study	T1: 3–8 weeks after surgery	Subjective data collection: Semi-structured interviews, initiated with: ‘‘Please tell me how you experienced being counselled to stop smoking before your BC surgery.’’
		**Age:** Median age = 50 (range 40–72)	**Stage**: Not reported	**Attrition/adherence:** Not reported			
		**Gender:** Female: *n* = 11	**Treatments:** Unresolved at time of interview: *n* = 2 Radiation therapy: *n* = 3 Endocrine therapy: *n* = 1 CT: *n* = 1 RT + CT: *n* = 1 RT + CT + endocrine therapy: *n* = 3				
Thomsen et al. 2010 Denmark [47]	Examine if a brief smoking cessation intervention encouraging patients to stop smoking from two days before to ten days after BC surgery would reduce the frequency of postoperative clinical complications requiring treatment	**Sample size:** *n* = 130 (I: *n* = 65, C: *n* = 65)	**Type:** Not reported	**Response rate:** *n* = 347 approached (*n* = 217 excluded), *n* = 130 consented, *n* = 113 analysed (I: *n* = 55, C: *n* = 58) Allocation: ×××I: *n* = 7 excluded (*n* = 6 withdrew prior to receiving intervention, *n* = 1 incorrect inclusion) ×××C: *n* = 3 excluded (*n* = 3 withdrew) Follow up: ×××I: *n* = 3 lost to follow-up (*n* = 3 withdrew) ×××C: *n* = 4 lost to follow-up (*n* = 3 withdrew, *n* = 1 death)	Single-blind randomised controlled multicentre trial	Baseline: at inclusion	Subjective data: Smoking cessation
		**Age:** I: 57.5 (35–79) years C: 56.5 (36–82) years	**Stage:** Not reported	**Attrition/adherence:** Not reported		T1: 2–10 days prior to surgery.	Objective data: Postoperative complications, length of hospital stay, requirement of secondary surgery, hospital readmission due to complication of primary surgery; exhaled carbon monoxide
						T2: 10 days post-surgery	
						T3: 30 days post-surgery	
		**Gender:** Female: *n* = 130	**Treatments:** BCS without axillary resection: I: *n* = 27 (47%), C: 32 (52%) BCS with axillary dissection: I: *n* = 14 (24%), C: *n* = 10 (16%) Mastectomy without axillary dissection: I: *n* = 7 (12%), C: *n* = 11 (18%) Mastectomy with axillary: I: *n* = 9 (15%), C: *n* = 9 (14%) Axillary dissection not complete: I: *n* = 1 (2%), C: *n* = 0			T4: 3 months post-surgery	
						T5: 6 months post-surgery	
						T6: 12 months post-surgery	
Tian et al. 2020 China [48]	Determine the influence of comprehensive nursing on the prognosis of BC patients, aiming to provide a better theoretical reference for future clinical nursing interventions with BC patients	**Sample size:** *n* = 168 (I: *n* = 98, C: *n* = 70)	**Type:** Not reported	**Response rate:** *n* = 168 consented	Controlled trial	T1: before treatment	Subjective data: MMSE; QOL; SAS; SDS; VAS; therapeutic effects; adverse reactions; nursing satisfaction
		**Age:** I: 42.3 ± 6.6 years C: 43.2 ± 7.5 years	**Stage:** Stage I–II: I: *n* = 78 (79.59%), C: *n* = 51 (72.86%) Stage III-IV: I: *n* = 20 (20.41%), C: *n* = 19 (27.14%)	**Attrition/adherence:** Not reported		T2: after treatment	Objective data: RECIST
		**Gender:** Not specified	**Treatments:** Not reported				
Wu et al. 2021 UK [49]	Assess the feasibility of multimodal prehabilitation as part of the BC treatment pathway	**Sample size:** *n* = 61	**Type:** Not reported	**Response rate:** *n* = 75 approached, *n* = 61 (81.3%) consented, *n* = 27 completed study, *n* = 24 (32%) completed all results, *n* = 20 nonparticipating patients consented to C Reasons for nonparticipation: surgery within 2 weeks (*n* = 14), full time commitments (*n* = 12), transportation difficulties (*n* = 8)	Prospective, cohort observational study	Baseline: preoperative	Subjective data collection: SF-12, HADS, SPADI
		**Age:** Median age = 63 (range 30–86) years	**Stage**: Not reported	**Attrition/adherence:** *n* = 12 attended 1–3 session, *n* = 12 attended ≥ 4 sessions		T1: 6 weeks post-surgery	Objective data collection: Usage of healthcare resources (length of stay and complications)
		**Gender:** Female: *n* = 61	**Treatments:** Surgery (not specified)				
Zgâia et al. 2016 Romania [50]	Investigate the effects of preoperative relaxing technique and psychological counselling on the postoperative intensity of acute pain, analgesic consumption and psychological symptoms, for patients scheduled for MRM for BC	**Sample size:** *n* = 102 (I: *n* = 58, C: *n* = 44)	**Type:** Not reported	**Response rate:** *n* = 115 approached (*n* = 13 excluded; *n* = 7 refused to participate, *n* = 4 met exclusion criteria, *n* = 2 had surgery postponed), 102 consented Assigned: I: *n* = 58, C: *n* = 44	Prospective, randomised, open-labelled, controlled trial (allocation ratio 1:1)	Postoperative pain:	Subjective data: Pain intensity and intensity of psychological symptoms using NRS
		**Age:** I: 52.25 ± 12.23 years C: 59.04 ± 10.75 years	**Stage:** Not reported	**Attrition/adherence:** *n* = 6 in I refused intervention and were assigned to C		T1: 0 h post-surgery	Objective data: Height, weight, BMI, consumption of analgesic drugs
						T2: 2 h post-surgery	
						T3: 8 h post-surgery	
						T4: 12 h post-surgery	
						T5: 24 h post-surgery	
						T6: every 6 h on the first day post-surgery	
		**Gender:** Not specified	**Treatments:** MRM: *n* = 102			Postoperative psychological symptoms: T1: every 6 h in the 48 h post-surgery	

*ALND* axillary lymph node dissection, *ASA* American Society of Anaesthesiologists, *AUDIT-C* Alcohol Use Disorders Identification Test, *BCS* breast conserving surgery, *BCT* breast conservation therapy, *BF%* body fat percentage, *BMI* body mass index, *BPI* Brief Pain Inventory, *C* control group, *CES-D* Center for Epidemiological Studies Depression Scale, *CT* chemotherapy, *DASH* Disabilities of the Arm, Shoulder, and Hand Questionnaire, *DCIS* ductal carcinoma in situ, *DES-IV* Differential Emotions Scale-IV, *EORTC QLQ-C30* European Organization for Research and Treatment of Cancer Quality of Life Core Questionnaire-30, *FACT-F* Functional Assessment of Cancer Therapy—Fatigue Questionnaire, *GLTEQ-LSI* Godin-Shephard Leisure Time Exercise Questionnaire – Leisure Score Index, *HADS* Hospital Anxiety and Depression Scale, *I* intervention group, *IDC* invasive ductal carcinoma, *IES* Impact of Event Scale, *IFN-γ* interferon-gamma, *ILC* invasive lobular carcinoma, *IQR* interquartile range, *LOT* Life Orientation Test, *MMSE* Mini-Mental State Examination, *MRM* modified radical mastectomy, *NK* natural killer, *NRS* numerical rating scale, *PSS* Perceived Stress Scale, *QOL* quality of life, *QoR-15* quality of recovery, *RECIST* Response Evaluation Criteria in Solid Tumours, *ROM* range of motion, *RT* radiotherapy, *sAA* salivary alpha-amylase, *SAS* Self-Rating Anxiety Scale, *SDS* Self-Rating Depression Scale, *SF-12* 12-Item Short Form Health Survey, *SF-36* 36-Item Short Form Health Survey, *SGPALS* Saltin-Grimby Physical Activity Scale, *SLNB* sentinel lymph node biopsy, *SPADI* Shoulder Pain and Disability Index, *SPOFIA* Subjective Perception of Post-Operative Functional Impairment of the Arm, *STAI* State-Trait Anxiety Inventory, *ULDQ* Upper Limb Disability Questionnaire, *VAS* Visual Analogue Scale, *WC* waist circumference, *WHODAS* 36-Item World Health Organization Disability Assessment Schedule 2.0, *6MWT* 6-min walk test, *7-Day PAR* 7-Day Physical Activity Recall

### Quality appraisal results

The results of the quality appraisal of the articles are presented in Table 2, where the quality assessment was carried out by all authors during the data extraction phase, and a second author then quality checked assessments on all articles, discussing any disagreements. After this process, it was found that all studies reported a generally low risk of bias. All studies reported a low risk of bias for the first two domains, which describe the outcomes of the studies addressing the research questions. Both the qualitative [46] and mixed-methods [38] studies reported a low risk of bias across all domains. For the quantitative studies, all groups were found to be comparable at baseline, and the reported outcome data were complete. An unclear bias risk was observed for some RCTs for outcome assessors being blinded to the intervention provided [40, 41, 44], and for participants adhering to the assigned interventions [45, 47], where this information was not described within the articles. Further, unclear bias was reported for all non-randomised quantitative studies [37, 42, 43, 48, 49] due to no discussion of accounting of confounders in the study design. A high bias risk was reported for a single study [39], as they stated that the study was not blinded to assessors, and the participants did not adhere to the intervention. Finally, while it is not defined as a quality criteria, it should be noted that one randomised study did not include a control group, but rather an additional intervention which acted as the comparator [40].

**Table 2:** Results of quality assessment

1. Qualitative	Item number of check list						
	S1	S2	1.1	1.2	1.3	1.4	1.5
Thomsen et al. [46]	Y	Y	Y	Y	Y	Y	Y
Item number check list key*: S1. Are there clear research questions, S2. Do the collected data allow to address the research questions, 1.1. Is the qualitative approach appropriate to answer the research question, 1.2. Are the qualitative data collection methods adequate to address the research question, 1.3. Are the findings adequately derived from the data, 1.4. Is the interpretation of results sufficiently substantiated by data, 1.5. Is there coherence between qualitative data sources, collection, analysis, and interpretation							

2. Quantitative Randomised Controlled Trials	Item number of check list						
	S1	S2	2.1	2.2	2.3	2.4	2.5
Heiman et al. [39]	Y	Y	Y	Y	Y	N	N
Knoerl et al. [40]	Y	Y	Y	Y	Y	U	Y
Larson et al. [41]	Y	Y	U	Y	Y	U	Y
Tamaki et al. [44]	Y	Y	Y	Y	Y	U	Y
Tanaka et al. [45]	Y	Y	Y	Y	Y	Y	U
Thomsen et al. [47]	Y	Y	Y	Y	Y	Y	U
Zgâia et al. [50]	Y	Y	Y	Y	Y	Y	Y
S1. Are there clear research questions, S2. Do the collected data allow to address the research questions, 2.1. Is randomisation appropriately performed, 2.2. Are the groups comparable at baseline, 2.3. Are there complete outcome data, 2.4. Are outcome assessors blinded to the intervention provided, 2.5. Did the participants adhere to the assigned intervention							

3. Quantitative Non-Randomised	Item number of check list						
	S1	S2	3.1	3.2	3.3	3.4	3.5
Baima et al. [37]	Y	Y	Y	Y	Y	U	Y
Sato et al. [42]	Y	Y	Y	Y	Y	U	Y
Springer et al. [43]	Y	Y	Y	Y	Y	U	Y
Tian et al. [48]	Y	Y	Y	Y	Y	U	Y
Wu et al. [49]	Y	Y	Y	Y	Y	U	Y
S1. Are there clear research questions, S2. Do the collected data allow to address the research questions, 3.1. Are the participants representative of the target population, 3.2. Are measurements appropriate regarding both the outcome and intervention (or exposure), 3.3. Are there complete outcome data, 3.4. Are the confounders accounted for in the study design and analysis, 3.5. During the study period, is the intervention administered (or exposure occurred) as intended							

5. Mixed Method	Item number of check list						
	S1	S2	5.1	5.2	5.3	5.4	5.5
Brahmbhatt et al. [38]	Y	Y	Y	Y	Y	Y	Y
S1. Are there clear research questions, S2. Do the collected data allow to address the research questions, 5.1. Is there an adequate rationale for using a mixed-methods design to address the research question, 5.2. Are the different components of the study effectively integrated to answer the research question, 5.3. Are the outputs of the integration of qualitative and quantitative components adequately interpreted, 5.4. Are divergences and inconsistencies between quantitative and qualitative results adequately addressed, 5.5. Do the different components of the study adhere to the quality criteria of each tradition of the methods involved							

*Three levels of assessment quality scores

*Y* Yes, *U* Unclear, *N* No

### Prehabilitation findings overview

A wide variety of prehabilitation interventions were completed for the included studies, as described in Table 3. For the selected studies, the majority completed exercise programs [37, 38, 40, 43, 49] or had a component of exercise [39, 42, 48], with two studies focusing on upper-limb exercise specifically [37, 38]. An additional two focused on smoking cessation [46, 47], with a single study reporting on multimodal prehabilitation [49], and a range of complementary and alternative therapies [41, 44, 45, 48, 50]. The program delivery methods varied and ranged from the education of recommended exercise to fully supervised exercise interventions and included the provision of relaxation, psychological, and educational programs. The duration of the interventions varied from a one-off 90-min session to 40-min sessions 3–5 times a week. An overview of the findings of these interventions can be found in Table 4.

**Table 3:** Overview of prehabilitation interventions

Author/Year	Purpose	Intervention
Baima et al. 2017 USA [37]	Explore the feasibility of an independent home shoulder exercise program to improve ipsilateral shoulder pain and abduction ROM after BC surgery	**Group 1 (in-person teaching):** Received an information sheet with exercises, video links and instruction with physical demonstration performed by the research team. The first exercise (Codman’s exercise) involves leaning over and tracing circles with the affected arm. The second exercise (scapular squeezes), involves standing with the arms above the head and pulling the elbows back and in. The third exercise (reach for the pillow) involves raising one arm above the head to reach pillows behind the head while lying supine
		**Group 2 (video only):** Received the instruction sheet only, with a link to the video as well as the recommended number of repetitions. The subjects were instructed to do the exercises daily before surgery and to stop at the time of surgery. They were restricted from any upper arm exercise above 90° of abduction while drains were in. They could resume the same exercises as desired after surgery and after all drains were removed
Brahmbhatt et al. 2020 Canada [38]	Determine the feasibility and acceptability of an individualised, home-based prehabilitation intervention prior to BC surgery	**Intervention:** Comprised of individually tailored, home-based exercise programs. Exercise prescriptions were developed and delivered by a RKin and consisted of aerobic exercise 3–5 days/week for 30–40 min per session, before and upper-quadrant-specific resistance training 2–3 days per week. Aerobic exercise prescriptions typically included brisk walking at an intensity of four-six on a 10-point RPE scale. Upper quadrant-specific resistance training consisted of two to three sets of 10–12 repetitions per exercise, with each session incorporating up to eight exercises (standing rows, shoulder external rotation, front raise, lateral raise, bicep curls, triceps extensions, wall push-ups, and chest press). Intervention also included stretching and mobility exercises which reflected standard postoperative rehabilitation. Participants were also provided with resistance bands and an exercise manual to facilitate home-based exercise. The RKin communicated with the participants on a weekly basis via phone calls or emails to support program compliance and appropriate progression and address any barriers to exercise (including questions about appropriate exercise completion) that may have prevented ongoing participation
Heiman et al. 2021 Sweden [39]	Evaluate whether an intervention consisting of recommended physical activity before and after surgery improved physical recovery at 4 weeks after BC surgery	**Intervention:** Recommended physical activity (add 30 min medium intensity aerobic activity daily) before and 4 weeks after surgery and completed questionnaires to track physical recovery
		**Control:** Standard care
Knoerl et al. 2022 USA [40]	Explore the impact of exercise and mind–body prehabilitation interventions on changes in quality of life and cancer treatment–related symptoms in women with newly diagnosed BC	**Intervention:** Participants were asked to attend two 60–90 min supervised exercise sessions with a certified trainer per week. Exercise intervention included both aerobic and resistance exercise training. Target exercise goals included 40 min of strength training and 180 min moderate intensity aerobic training each week
		**Control:** The mind–body control participants were given a book and asked to listen to an associated guided imagery audio guide twice a day
Larson et al. 2000 USA [41]	Evaluate the feasibility and potential immunological benefit of a presurgical psychosocial intervention for BC patients	**Intervention:** Participants attended two 90-min treatment or “intervention” sessions. Both sessions included psychosocial support including discussion about emotional impacts of initial diagnosis and impending surgery, identification of problems/difficulties warranting further attention, education about the impact of stressful life events on health, developing individually tailored problem-solving strategies before and an introduction to progressive muscle relaxation exercises. Both sessions concluded with participant debriefing and completion of a feedback questionnaire
		**Control:** Standard care
Sato et al. 2014 Japan [42]	Investigate the effectiveness of a perioperative education program for improving upper arm dysfunction in patients with BC	**Intervention:** Received preoperative education regarding the mechanism and causes of symptom development; postop, they were taught techniques to prevent/improve impairment, including monitoring symptoms, exercises, and massage techniques; individual support provided between 1 and 3 months to enhance symptom management
		**Control:** Standard care
Springer et al. 2010 USA [43]	Determine the extent and time course of upper-limb dysfunction in subjects seen pre-operatively and followed prospectively using a novel physical therapy surveillance model post-BC and treatment	**Intervention:** Participants were instructed in a postoperative upper-limb ROM exercise program and educated regarding upper-limb lymphedema precautions and physical exercise initiation and progression. The exercise program was reviewed 1-month post-surgery, and individualised home program instructions provided as needed
Tamaki et al. 2017 Japan [44]	Compare the effects of aromatherapy on mood, quality of life, and physical symptoms in patients with BC	**Intervention:** Participants had aroma oil placed at the bedside from 9 pm of the day before surgery day until 6 am of the surgery day. The aromatherapy consisted of the choice of three kinds of aroma oil, including ylang-ylang, orange or lavender
		**Control:** Standard care
Tanaka et al. 2021 Japan [45]	Assess the effects of Yokukansan on BC patients undergoing a practical or total mastectomy breast surgery	**Intervention:** Received two 2.5 g doses of the medication (Yokukansan) before sleeping the night before surgery and 2 h before induced anaesthesia
		**Control:** did not receive the medication
Thomsen et al. 2009 Denmark [46]	Explore how women smokers with newly diagnosed BC experienced brief preoperative smoking cessation intervention in relation to BC surgery	**Intervention:** Smoking intervention took place three to seven days before surgery and consisted of one counselling session lasting 45–90 min with trained smoking cessation counsellors. The principles of motivational interviewing inspired the intervention. Content entailed that the risks of smoking and the health benefits of smoking cessation in relation to surgery and in the long-term were discussed with participants. Qualitative interviews were conducted by a single author 3–8 weeks after surgery, with interviews lasting from 35 to 100 min
Thomsen et al. 2010 Denmark [47]	Examine if a brief smoking cessation intervention encouraging patients to stop smoking from two days before to ten days after BC surgery would reduce the frequency of postoperative clinical complications requiring treatment	**Intervention:** Smoking intervention took place three to seven days before surgery and consisted of one counselling session lasting 45–90 min with trained smoking cessation counsellors. Additionally, NRT was offered for the recommended perioperative smoking cessation period
		**Control:** Standard care
Tian et al. 2020 China [48]	Determine the influence of comprehensive nursing on the prognosis of BC patients, aiming to provide a better theoretical reference for future clinical nursing interventions with BC patients	**Intervention:** Comprehensive Nursing Intervention: Medical staff engaged in hospital education for participants and their families to improve their correct understanding of BC diseases and described successful cases to enhance the confidence of participants and families. They also strictly required participants to eat a healthy diet and gave effective guidance to participants and their families. Health care staff strictly required participants to exercise their upper limbs and provided professional guidance to ensure a balanced diet. They also urged participants to engage in appropriate outdoor exercise and to keep an optimistic attitude. If the participants had adverse reactions, they were appeased right away, and certain methods were adopted to improve participants’ discomfort and prognosis
		**Control:** Conventional Nursing Mode: Nurse instructed participants in the rational administration of the drug, explained the disease to participants, performed assigned tasks, including infusions, vital sign monitoring, gave appropriate psychological counselling, life guidance, and other nursing interventions
Wu et al. 2021 UK [49]	Assess the feasibility of multimodal prehabilitation as part of the BC treatment pathway	**Intervention:** Received four types of intervention (supervised exercise, nutrition, smoking cessation, and psychosocial support: --Supervised exercise consisted of two circuits (circuit one: sit-to-stand, horizontal row, calf raises and chest press; circuit two: deadlift, pullover, knee extension, shoulder press) which were both performed three times a session, with each exercise repeated 8–12 times --Nutritional advice consisted of the consumption of adequate amounts of protein and consider the nutrient density of the foods they consume --Smoking cessation provided participants with necessary therapies and advice to help them quit smoking, including NRT, patches, and gum --Psychological support was assigned if participants identified with raised anxiety and/or depression scores. Participants had at least one session with their counsellor as part of their program
		**Control:** Standard care
Zgâia et al. 2016 Romania [50]	Investigate the effects of pre-operative relaxing technique and psychological counselling on the postoperative intensity of acute pain, analgesic consumption and psychological symptoms, for patients scheduled for MRM for BC	**Intervention:** Received 50 min of relaxation technique in the morning of the surgery. This included 25 min of a short clinical semi-structured interview regarding history of the disease and treatment of the participants, offering supplementary information regarding the surgery and its complications, and 25 min of autogenous training exercise known as “autogenous training” or “Schultz relaxation method” combining visual imagery and suggestions to experience relaxation and peace
		**Control:** Did not receive any psychological intervention before surgery

*MRM* modified radical mastectomy, *NRT* Nicotine Replacement Therapy, *RKin* Registered Kinesiologist, *ROM* range of motion, *RPE* Rating of Perceived Exertion, *sAA* salivary alpha-amylase

**Table 4:** Overview of study findings

Author/Year	Study outcomes	Physical function assessments	Clinical assessments	Patient reported outcome measurement	Findings
Baima et al. 2017 USA [37]	Primary outcome: 11-point pain scale (0–10), ROM (0–180°), and chart documentation of postoperative seroma formation	11-point pain scale (0–10), ROM (0–180°)	Chart documentation of postoperative seroma formation	Not assessed	Data failed to provide strong evidence of a difference in exercise compliance between in-person teaching versus video teaching. (75%, 24/32 vs. 77%, 10/13, OR = 1.03)
					Sixty-six per cent of participants (20/30) lost greater than 10° shoulder abduction ROM at 1-month post-surgery
					29% of participants (9/31) had worse shoulder pain than baseline at 1-month post-surgery (24%, 6/25 exercisers, and 50%, 3/6 non-exercisers)
					Fifteen percent of participants (4/27) had pain worse than baseline at 3 months post-surgery (8%, 2/25 exercisers, and 100%, 2/2 non-exercisers)
					Strength data did not show significance
Brahmbhatt et al. 2020 Canada [38]	Primary and secondary outcomes not clearly reported Quantitative feasibility outcomes, qualitative assessment of feasibility, and participant experience	6MWT; upper-extremity strength (handgrip dynamometry); manual muscle testing (digital handheld dynamometer); WC, BMI, lean body mass, BF%, and fat mass	Clinical disease related data collected from chart review	DASH; BPI; FACT-F; SF-36 v2; GLTEQ-LSI; WHODAS 2.0	The 6MWT distance increased from baseline to the preoperative assessment by 57.10 ± 24.0 m (95% CI 7.52, 121.7)
					Small decrease in 6MWT distance from the preoperative assessment to the 6-week postoperative assessment [− 5.51 ± 27.6 m (− 79.74, 68.7)], scores remained greater than at baseline
					Increase in 6MWT distance of 62.90 ± 24.00 m (1.81, 127.60) from baseline to the last study assessment
					An increase in DASH scores of 16.18 ± 4.96 (2.74, 29.63) points was observed between the preoperative and 6-week postoperative assessment, indicating a clinically important increase in upper-quadrant disability (MCID of 15 points)
					Overall worsening in fatigue levels from baseline to the 12-week postoperative assessment, demonstrated by a reduction of 4.63 ± 3.34 (− 13.7, 4.41) points in FACT-F scores which have an MCID of three points
					SF-36 questionnaire consistently worsened over the study period with a decrease of 5.90 ± 2.17 (-11.75, -0.05) points from the first to the last assessment
					SF-36 mental component score worsened from baseline to the preoperative assessment but then improved by 4.36 ± 2.25 (− 1.72, 10.44) points from the pre- to 6-week postoperative assessment
					GLTEQ-LSI scores increased over the study period from 22.8 ± 5.30 at baseline to 33.8 ± 6.12 at the last study assessment
					Qualitative subthemes: Intervention feasibility: appropriateness of the intervention, barriers, and facilitators to participation. Participants’ experiences: intervention design preferences, multimodal care, need for an exercise professional, perceived benefit, health behaviour change, regaining control, prehabilitation as education
Heiman et al. 2021 Sweden [39]	Primary outcome: physical recovery at 4 weeks after surgery, measured using self-administered questionnaires Secondary outcomes: self-reported mental recovery at 4 weeks after surgery	Not assessed	Medical records were accessed to obtain surgical information	SGPALS; AUDIT-C	There was no significant difference in favour of the intervention for the primary outcome physical recovery (RR = 1.03, 95%; CI 0.95–1.13)
					There was also no difference for mental recovery (RR = 1.05, 0.93–1.17) nor in mean Comprehensive Complication Index score [4.2 (range 0–57.5) versus 4.7 (0–58.3)] between I and C
					64.1% in I did not report any change in physical activity level; 66/0% in C
Knoerl et al. 2022 USA [40]	The impact of exercise and mind–body prehabilitation intervention on changes in quality of life and cancer treatment-related symptoms	7–Day PAR	Clinical chart and diagnosis	EORTC QLQ C30; PSS; HADS	Significant improvements in exercise amount for I: 203 ± 129 min/week, compared to C: 23 ± 76 min/week (*P* < 0.0001)
					Mind–body group participants experienced significant improvements in cognitive functioning in comparison to exercise group participants between T1 and T3
					Difference in average change: − 9.61, *P* = 0.04, *d* = 0.31
					Both groups experienced improvement in anxiety (exercise: average change = − 1.18, *P* = 0.03, *d* = 0.34, mind–body: average change = − 1.69, *P* = 0.006, *d =* 0.43) and perceived stress (Stress improvement: Exercise: average change = − 2.33, *P* = 0.04, *d =* 0.30, mind–body: average change = − 2.59, *P* = 0.05, *d =* 0.29)
					Improvements were seen in the mind–body group for insomnia (average change = − 10.03, *P* = 0.04, *d =* 0.30) and cognitive function (average change = 13.16, *P* = 0.0003, *d =* 0.67)
					Both groups experienced a significant decline in role functioning (exercise: average change = − 11.10, *P* = 0.005, *d =* 0.43; mind–body: average change = − 11.31, *P* = 0.009, *d =* 0.40) over time
					No significant changes (*P* > 0.05) in physical function, fatigue, or pain
Larson et al. 2000 USA [41]	Evaluate whether BC patients who participated in the psychosocial intervention would have improved immune function, as measured by an increase in both NK cell activity and IFN-γ production, and whether responses to psychological (i.e. self-report) measures would mirror these immune system shifts	Immune function via NK cell activity and IFN-γ production	Not assessed	CES-D, DES-IV, IES, LOT, SF-36	Analysis of NK cell activity did not yield any significant differences between the control and the experimental groups. Results did not support the idea that the presurgical psychosocial intervention in any way influenced the NK cell response
					IFN-γ levels decreased substantially over time in C but not for I, suggesting that the intervention may have been successful in reducing immunosuppression prior to surgery. However, this finding is clouded by no significant difference in IFN-γ levels at the baseline timepoint
					Participants in I showed a decrease in feelings of cancer-related disgust over time, whereas control participants experienced an exacerbation of those same feelings
Sato et al. 2014 Japan [42]	Evaluate provision of perioperative exercise program vs standard care, evaluate the subjective perception of postoperative functional impairment and disability of the arm and shoulder, in combination with objective measurements of measured arm girth, shoulder ROM, and grip strength	Arm girth; shoulder ROM; grip strength	Medical records provided notes on type of surgery, level of ALND, and adjuvant treatment	SPOFIA; DASH	SPOFIA and grip strength were significantly improved in the I who underwent ALND; no significant improvement in the I who underwent SLNB
					No significant differences in arm girth, shoulder ROM, or DASH were seen between groups with ALND. Significant differences in change in SPOFIA score over time were noted between the ALND I and C groups (*F* value = 3.34; *P* = 0.02)
					A significant difference over time in the difference in mean grip strength was seen between normal and affected sides in both I and C groups (*F* value = 2.77; *P* = 0.04), indicating significantly improved grip strength over time in the ALND I group compared to the ALND C group
					No significant differences in arm girth, shoulder ROM, grip strength, SPOFIA, or DASH were identified between SLNB groups
Springer et al. 2010 USA [43]	Primary outcome: the extent and time course of upper-limb dysfunction in subjects seen pre-operatively and followed prospectively using a novel physical therapy surveillance model post-BC and treatment Secondary outcomes: determine if pain is a factor in recovery and assess self-report of functional task difficulty 12 months post-surgery using ULDQ	Shoulder ROM (flexion, abduction, internal rotation, and external rotation) and strength; upper-limb volume and girth	Not assessed	ULDQ	Shoulder abduction, external rotation, flexion, and composite ROM decreased from baseline to 1 month (*P* < 0.0001), improved from 1 month to 3–6 months (*P* < 0.0001), and improved further from 3–6 to 12 months (*P* < 0.0001)
					Internal rotation ROM had a significant decrease from baseline to 1 month (*P* < 0.04), and a significant improvement from 1 and 3–6 months to 12 months (*P* < 0.03)
					Shoulder strength had a significant decrease at 1 month (*P* < 0.001)
					Greater pain at 1 month than baseline (*P* < 0.001)
					Shoulder abduction, flexion, external rotation, and composite ROM significantly correlated with all subcategories of the ULDQ (*P* < 0.02); internal rotation did not
					Significant difference in limb volume found between sub-clinical lymphedema and no lymphedema subgroups at 12 months (*P* < 0.045)
Tamaki et al. 2017 Japan [44]	Primary endpoint: QOL, which was assessed EORTC QLQ-C30 Secondary endpoints: necessity of hypnotics, vital signs (blood pressure and heart rate), adverse events, and patient perception of the experience	Vital signs (blood pressure and heart rate)	Not assessed	EORTC QLQ-C30; use of hypnotics; patient experience	No statistically significant differences between groups in the EORTC QLC-C-30 at the surgery day. Differences in physical functioning and role functioning detected for post-operation day 1 but did not reach statistical significance (*P* = 0.08 and 0.09, respectively)
					No effects of aromatherapy were observed for blood pressure, heart rate, and the rate of hypnotic using (all *P* > 0.05)
					Patient experience was positive (participants were relaxed, comfortable, and enjoyable)
Tanaka et al. 2021 Japan [45]	Primary outcome: changes in sAA as an objective measure of anxiety Secondary outcomes: subjective measures of anxiety through HADS, STAI, QoR-15, and VAS for pain intensity	sAA levels were monitored pre- and post-surgery as an anxiety indicator	Clinical charts and BC diagnosis	HADS (HADS-A and HADS-D); STAI (STAI-S and STAI-T); QoR-15; VAS for pain intensity	Difference in HADS-A: I: − 2.77, 95% CI [− 1.48–− 4.06], *P* < 0.001, and C: − 1.43 [− 0.25–− 2.61], *P* = 0.011
					Difference in STAI-T: I: mean − 4.23 [− 6.95–− 1.51], *P* = 0.0004: and C: 0.12 [− 2.36–− 2.60], *P* = 0.92
					sAA scores significantly lower in I group at T2: I: 0.88 (0.42); C: 1.14 ± 0.49: F [2, 150] = 3.76; *P* = 0.03; gη^2^ = 0.017
					No significant differences in HADS-D, STAI-S, and VAS scores between groups
Thomsen et al. 2009 Denmark [46]	Qualitative experiences related to the prehabilaition smoking intervention	Not assessed	Not assessed	Not assessed	Reflecting upon smoking and health Participants used metaphors such as ‘‘the final push,’’ ‘‘a kick’’ and ‘‘a wake-up call’’ to describe their experience of being offered brief preoperative smoking intervention. Major motivation for wanting to stop smoking was the risk of postoperative wound healing complications “‘It’s a combination. Being told you have cancer. Of course, BC hasn’t got anything to do with smoking. That’s more lung cancer but you begin to think in other directions. How much at risk are you of getting other types of cancer? And that’s why I thought: No, I’ve already got asthma, so I have to quit. And being offered smoking intervention before surgery gave me the last push.’’
					Escaping the social stigma of being a smoker Described by the participants as increasingly awkward due to restrictive smoking policies and disapproval of smoking ‘‘Shortly before [being offered the smoking intervention] I told her [my daughter] that I had cancer, she said to me oh mum will you please stop smoking. You know I’d like to and I’ve wanted to and I have also tried twice. Just don’t ask me to do it now. Right now, I can’t face it with the cancer and all that. But you know I came to a point, yes, because I think it’s easier to be a non-smoker.”
					Heightened awareness of being addicted to smoking Some participants experienced that the smoking intervention motivated smoking cessation for a short period before or immediately after surgery; however, they quickly resumed their habit, or alternatively, attempted but were unable to stop smoking at all Specifically, anxiety peaked in the days prior to surgery and to getting the results of surgery. Brief preoperative smoking intervention in this context did not sustain abstinence in these participants ‘‘Absolutely, I’m never going to smoke another cigarette, ever again. But going through the hospital system and all the waiting and pressure and worrying. Then you start to think: If I smoke just one cigarette here and another one there, it won’t harm anything, because you need it.’’
					Enacting a duty of responsibility The participants who stopped smoking experienced doing so as an enactment of a duty of responsibility towards themselves and those nearest to them ‘‘I want to be well again and live a healthy life. The most important thing is to be able to be here for my kids and not to pollute them with my smoking.’’
Thomsen et al. 2010 Denmark [47]	Primary objective: Postoperative complications, defined as death or postoperative morbidity requiring treatment within 30 days after surgery (including seroma requiring aspiration) Secondary objectives: self-reported smoking cessation (two days before to ten days after surgery), exhaled carbon monoxide, and long-term continuous smoking cessation	Not assessed	Patients’ charts examined for clinical complications	The Fagerström Test for Nicotine Dependency Score; smoking diary; telephone interviews for long-term cessation	Brief smoking intervention increased self-reported perioperative smoking cessation without having any clinical impact on postoperative complications
					Significantly more I participants (16/57; 28%) than C participants (7/62; 11%) reported continuous abstinence from two days before to ten days after surgery (RR = 2.49; 95% CI 1.10–5.60)
					At 12 months, groups did not differ in smoking cessation (I: 7/55, 13%; C: 5/58, 9%; RR = 1.48; 95% CI 0.50–4.38)
					Median carbon monoxide levels on day of surgery were significantly different: I: 2 ppm (range: 0–33); C: 4 ppm (range 0–36); p-0.04)
					Carbon monoxide levels did not differ significantly between groups after ten days: I: 5 ppm (range: 0–43); C: 6.5 ppm (range: 0–52); *P* = 0.14
					Postoperative complications (I: 52%; C: 64%; *P* = 0.35) and wound complications (I: 44%; C: 47%; *P* = 1.00) within 30 days did not differ
Tian et al. 2020 China [48]	Primary outcomes: observe the clinical curative effect of the two groups, and compare the therapeutic effects, adverse reactions, nursing satisfaction, VAS pain, psychological state SAS and SDS, and QOL of the two groups Secondary outcomes: compare the MMSE of the groups	Target lesions evaluated according to RECIST Pain evaluated according to VAS scores	Not assessed	QOL; SAS; SDS; MMSE	Curative effect: No difference in total effective rate between the two groups (*P* = 0.400)
					No differences in the adverse reactions in the two groups (*P* > 0.05)
					Number of participants who were very satisfied with nursing in I was higher than C (*P* < 0.05)
					VAS scores in I after treatment (2.45 ± 1.26) were significantly lower (*P* < 0.05) than C scores (3.73 ± 1.39)
					SAS and SDS scores significantly lower in I than C after treatment, and scores significantly lower in both groups after treatment than baseline (all *P* < 0.05)
					MMSE scores in I significantly higher than in C after treatment (*P* < 0.05)
					QOL scores in I higher than in C (*P* < 0.05)
					No differences in participant survival in the two groups (*P* > 0.05)
Wu et al. 2021 UK [49]	Feasibility was determined by the multimodal prehabilitation compliance	Not assessed	Length of inpatient stay, hospital readmissions and complications	SF-12; HADS; SPADI	Anxiety scores (HADS) were significantly lower after surgery in both groups participating in prehabilitation (1–3 sessions: *P* = 0.028; ≥ 4 sessions: *P* = 0.045). The remaining outcomes analysed did not demonstrate significant changes (*P* > 0.05)
					Median length of stay was 2 days/1night for both prehabilitation cohorts. No 30-day complications requiring further hospitalisation and no hospital readmissions recorded
Zgâia et al. 2016 Romania [50]	Primary outcome: Postoperative pain intensity Secondary outcomes: presence and intensity of psychological symptoms; analgesic consumption (Opioids, Paracetamol, NSAIDs)	Not assessed	Not assessed	NRS (for pain intensity); NRS (for intensity of psychological symptoms)	Pain intensity significantly lower in I than C immediately after waking, at T2, T3, T4, and T5 after surgery (*P* < 0.05). I recorded maximum pain intensity 4/10 NRS. C recorded maximum pain intensity 8/10 NRS. Difference between mean scores of groups approximately 5/10 NRS immediately after waking (I: 1.5/10; C: 6/10), and a difference of 3/10 NRS 2 h post-surgery (I: 1/10; C: 4/10)
					Amount of post-surgery intravenous opioid used (ampoules of 100 mg tramadol) and NSAIDs (ampoules of 100 mg ketoprofen) significantly lower in I compared to C, both on the first and the second day after surgery (both *P* < 0.001). Amount of intravenous paracetamol (bottles of 1 g paracetamol) similar in both groups
					Higher frequency of psychological postoperative symptoms in C compared to I during evaluation period. Differences statistically significant at 6 (*P* < 0.001), 12 (*P* = 0.011), 24 (*P* = 0.021), 30 (*P* = 0.006), and 48 (*P* = 0.021) hours after surgery, and not at 42 h (*P* = 0.537). At 48 h, intensity of psychological symptoms lower in I compared to C

*ALND* axillary lymph node dissection, *AUDIT-C* Alcohol Use Disorders Identification Test, *BF%* body fat percentage, *BMI* body mass index, *BPI* Brief Pain Inventory, *C* control group, *CES-D* Center for Epidemiological Studies Depression Scale, *CI* confidence interval, *DASH* Disabilities of the Arm, Shoulder and Hand Questionnaire, *DES-IV* Differential Emotions Scale-IV, *EORTC QLQ-C30* European Organization for Research and Treatment of Cancer Quality of Life Core Questionnaire-30, *FACT-F* Functional Assessment of Cancer Therapy—Fatigue Questionnaire, *GLTEQ-LSI* Godin-Shephard Leisure Time Exercise Questionnaire – Leisure Score Index, *HADS* Hospital Anxiety and Depression Scale, *HADS-A* Hospital Anxiety and Depression Scale-Anxiety, *HADS-D* Hospital Anxiety and Depression Scale-Depression, *I* intervention group, *IES* Impact of Event Scale, *IFN-γ* interferon-gamma, *LOT* Life Orientation Test, *MCID* minimal clinically important difference, *MMSE* Mini-Mental State Examination, *NK* natural killer, *NRS* numerical rating scale, *NSAIDs* nonsteroidal anti-inflammatory drug, *PSS* Perceived Stress Scale, *QOL* quality of life, *QoR-15* quality of recovery, *RECIST* Response Evaluation Criteria in Solid Tumours, *ROM* range of motion, *RR* risk ratio, *sAA* salivary alpha-amylase, *SAS* Self-Rating Anxiety Scale, *SDS* Self-Rating Depression Scale, *SF-12* 12-Item Short Form Health Survey, *SF-36* 36-Item Short Form Health Survey, *SGPALS* Saltin-Grimby Physical Activity Scale, *SLNB* sentinel lymph node biopsy, *SPADI* Shoulder Pain and Disability Index, *SPOFIA* Subjective Perception of Post-Operative Functional Impairment of the Arm, *STAI* State-Trait Anxiety Inventory, *STAI-S* State-Trait Anxiety Inventory (current), *STAI-T* State-Trait Anxiety Inventory (general), *ULDQ* Upper Limb Disability Questionnaire, *VAS* Visual Analogue Scale, *WC* waist circumference, *WHODAS* 36-Item World Health Organization Disability Assessment Schedule 2.0, *YKS* Yokukansan, *6MWT* 6-min walk test, *7-Day PAR* 7-Day Physical Activity Recall

#### Upper limb evaluation in prehabilitation

Five studies explored the effects of prehabilitation on upper-limb (UL) dysfunction following surgical treatment for BC [37, 38, 42, 43, 49]. Study sizes ranged from 28 to 162 participants, with all research time points commencing pre-operatively but ending at different time points (6 weeks, 12 weeks, and 12 months). The heterogeneity observed for the designs of these studies limits the understanding of the long-term impact of prehabilitation on UL dysfunction.

Participants partook in prehabilitation exercise programs and education, face to face or by video, and in some cases were compared to standard care with respect to UL dysfunction. Standard care was not necessarily discussed, compared, or explained in the research. Three studies [38, 42, 49] utilised the Disabilities of the Arm, Shoulder, and Hand (DASH) Questionnaire, measuring the ability of the participants to complete upper-extremity activities, which allowed consistency in results. The Shoulder Pain and Disability Index (SPADI), Upper Limb Disability Questionnaire (ULDQ), and Subjective Perception of Post-Operative Functional Impairment of the Arm (SPOFIA) were other tools utilised to measure UL functionality.

It was identified that UL pain [37] and DASH score [38] increased over time from baseline measurements, indicating an increase in UL disability. Similarly, there was a decrease in shoulder range of motion between baseline and measurements at one month; however, the range of motion improved at all subsequent time points [43]. While one study reported no significant changes between groups for SPADI [49], improved grip strength and SPOFIA score were reported in patients who had participated in prehabilitation and who had undergone axillary dissection [42]. However, no significant improvements were observed following prehabilitation in patients having sentinel node biopsy [42].

#### Exercise and physical activity programs (excluding UL only)

Two studies included a supervised exercise program within the BC prehabilitation intervention which included both aerobic and resistance training [38, 40], with one of these studies completing UL-specific resistance training [38]. These interventions consisted of 30–40 min aerobic exercise 3–5 days per week, with 2–3 UL resistance training days a week [38], and two 60–90 sessions a week incorporating both aerobic (30–40 min) and resistance (20 min) exercises [40] (Table 2). Both studies identified that disability increased from baseline to 12 weeks post-operatively [38], and a decrease in role functioning over time [40] despite the exercise programme. However, an increase in the six-minute walk test (6MWT) was observed from baseline to pre-operatively indicating an increase in aerobic fitness prior to surgery [38], with improvements made for anxiety and stress in the exercise group [40]. Interestingly, the interviews completed by Brahmbhatt et al. [38] revealed that the participants and the healthcare professionals were in favour of the program. Participants suggested that the programme is offered to all surgical candidates, as it helped them to regain control over the preoperative period and it facilitated postoperative recovery by educating on postoperative rehabilitation protocols. A preference for multimodal prehabilitation was highlighted, due to the request for the inclusion of dietetics and psychological interventions.

#### Complementary and alternative therapies

A range of studies provided complementary and alternative therapies as part of their prehabilitation interventions. These included psychosocial [41], aromatherapy [44], traditional medicine [45], comprehensive nursing [48], and relaxation technique [50] interventions, with another study utilising a mind–body prehabilitation program as a comparison to their prescribed exercise intervention [40]. The duration of these studies was short term and ranged from one day to one-month post-surgery, with sample sizes ranging from 41 to 168 participants. All but one study [48] was a randomised controlled trial, with the comparator groups completing a standard care that was unspecified by the authors [41, 44, 50]. This is with the exception of Tanaka et al. [45] who prescribed water to participants in place of the traditional medication, and Tian et al. [48], who described what the ‘Conventional Nursing Mode’ their control group received entailed. Further, Knoerl et al. [40] did not utilise a control, but rather two groups who received different interventions.

A range of patient-reported outcomes were assessed in these studies, with only one study reporting no significant differences between groups for any outcomes, resulting in aromatherapy potentially not being successful for prehabilitation in this population [44]. The mind–body intervention prescribed by Knoerl et al. [40] resulted in improvements in cognitive decline, however, also reported a significant decline in role functioning over time [40]. The comprehensive nursing intervention resulted in higher mental state and quality of life scores, and lower anxiety, depression, and pain scores [48], while participating in relaxation techniques and psychological counselling also resulted in lower pain intensity and significantly lower postoperative psychological symptoms [50].

To support these patient-reported outcomes, two studies collected additional information on the participant’s biomarkers. Larson et al. [41] reported a decrease in “cancer-related disgust”, and while there were no significant changes to natural killer cell activity, it was observed that interferon-gamma did not substantially decrease in the intervention group, suggesting that the psychosocial intervention may have played a role in preventing treatment-related immunosuppression [41]. Additionally, the prescription of traditional medicine significantly reduced Hospital Anxiety and Depression Scale-Anxiety (HADS-A) and State-Trait Anxiety Inventory-General (STAI-T) symptoms in the intervention group [45]. Tanaka et al. [45] reported significantly lower salivary alpha-amylase immediately prior to surgery, supporting the decreased patient-reported anxiety [45].

#### Cessation of smoking

Two publications reported on the same study that explored the cessation of smoking interventions before and after BC surgery [46, 47], reporting on both the qualitative [46] and quantitative [47] outcomes of the intervention. Participants were required to attend a single session of counselling and were provided with nicotine replacement therapy. The quantitative findings identified that more participants in the intervention arm reported continuous smoking cessation across a short-term surgery period [47]. Alternatively, no difference was observed between the control and intervention groups in postoperative complication rate, wound complication rate, or long-term smoking cessation [47]. Thomsen et al. [46] utilised semi-structured interviews for the qualitative aspect of this research and reported that the intervention encouraged the participants to reflect on their smoking, which encouraged short-term cessation for their surgery [46]. However, it was revealed that a prolonged intervention duration, both pre- and post-operatively may be more effective in supporting smoking cessation in this population [46].

#### Multimodal prehabilitation

One of the included studies explored the impact of multimodal prehabilitation in participants with BC [49]. The interventions included self-management topics related to nutrition, smoking cessation, psychosocial support, and a tailored exercise program [49]. Overall, 81% (*n* = 61) of participants with BC chose to participate, with *n* = 20 participants declining the multimodal prehabilitation intervention being assigned to the control group. Noteworthy, the researchers did not clearly define what the current standard of care was in the control arm, and therefore, bias is possible in study outcomes. Overall, there were no statistically significant differences in length of hospital stay, rates of readmissions, post-treatment complications or health-related QOL scores, with the exceptions of anxiety levels in favour of the intervention arm [49]. The maximum follow-up time point post-surgery was six weeks [49], which limits the understanding of the impact of multimodal prehabilitation into survivorship.

## Discussion

Prehabilitation is an emerging research area [51]. The time before treatment can be used to introduce self-management support to optimise recovery in individuals with BC. At present, there seems to be a narrow focus on the type of programs delivered post-treatment and variations in standard care, making it difficult to compare interventions and outcomes. Prehabilitation had a positive impact on health-related outcomes of participants diagnosed with BC, and one study reported reduced anxiety, stress, and insomnia scores [40]. It was identified that the prehabilitation period may be the ideal time period to determine the needs of individuals with BC [38]. Ensuring timely referrals would likely reduce fatigue, disability of the arm and hand, and improve overall psychological and physical fitness and strength post-operatively. The focus at present is on a single form of treatment/support which for most studies was lacking a holistic approach to the participant’s care and input from the muti-disciplinary team. There is an opportunity for improved clinical, psychological, physical, and quality of life outcomes with the implementation of prehabilitation in BC. This review determined that to date, the evidence in prehabilitation in BC is emerging.

Multi-modal prehabilitation should be considered by clinicians within clinical service re-design [23]. Given that there is only a short timespan between diagnosis and the first line of treatment, it is important to consider how the program would work and how the delivery of the different components could compliment the treatment. Given that there was only one multimodal intervention [49] within this review, it is difficult to draw conclusions on what this program should look like. The program was feasible with 80% of patients opted to participate and was found to reduce anxiety levels [49]. The follow-up period was only six weeks which means that long-term effects of the program cannot be determined. Future programs should consider a longer-term follow-up period in their study designs. Partners could also be an influence on the success of prehabilitation programs [33]; however, to date, there is limited evidence.

Multimodal prehabilitation should consider all facets of the person and include support from oncology specialist nurses and programs such as exercise, psychology, and nutrition and educational components as needed by the individual. Long-term physical and psychological wellness is key to recovering from BC treatments and its side effects. Implementing a prehabilitation program could ensure people are well supported as they go through this period of their lives.

Quitting smoking has been shown to increase survival, improve healing times and surgical outcomes [52] among BC patients, although it is not known what support is needed and when the best time to deliver it is [53]. Smoking cessation in this review focused on education and counselling with varying results. The support may be better integrated across the diagnosis and treatment phase when it is best for the patient, however, should form part of an early conversation with the patient.

As part of holistic care for participants with BC prehabilitation, UL assessment and therapy should form part of prehabilitation given that UL dysfunction is a common effect of treatment. UL pain was shown to increase over time indicating that disability will also increase for many. This shows the need to support the patient through the treatment phase and beyond even with a successful prehabilitation program. Understanding the impact of movement and exercise over this period should be a high priority and future direction for research. Exercise improved anxiety and stress and participants and health care workers were supportive of the program and believed it helped with their recovery. The qualitative exploration identified that participants wanted multimodal interventions rather than unimodal.

Complementary and alternative programs may be supportive of the goal of prehabilitation in people with BC. These included psychological support, aromatherapy, traditional medicine, comprehensive nursing, and relaxation techniques including mind–body work. Improvements in cognition, mental state, quality of life, decreased anxiety, depression, and pain were attributed to complementary therapies, in all but one study in this review.

While some significant changes were observed in this review there are limitations that must be considered. Studies did not always report the type of BC (or treatment/ specific surgery details), increasing the heterogeneity, and not allowing us to draw more accurate conclusions for the type of participants, including male participants. However, what we have shown is that prehabilitation could improve many outcomes for people with breast cancer and should be further explored.

### Clinical implications

This review makes an important contribution which has acknowledged for the first time that significant heterogeneity exists in prehabilitation models of care in terms of the mode of administration, duration, and outcome measures used to quantify its impact. Importantly, there has been a lack of focus on the outcomes of including partners as critical companions during this distressing phase of the cancer care continuum. Members of the multidisciplinary team caring for people affected by BC are encouraged to use the findings of this review to inform holistic models of care.

## Conclusion

Prehabilitation for patients with BC is an emerging research area that appears to improve outcomes; however, ensuring adequate intervention timeframes, follow-up, and population groups should be considered for future investigations. Researchers and healthcare professionals still do not know the contribution of the effect of unimodal influence on study outcomes compared to multimodal interventions, and what approach is most effective to optimise clinical, physical, and psychological outcomes.

### Future research

Based upon the findings of this review, future RCT multimodal prehabilitation programs are needed and considerations should be given to include partners. There was a lack of prospective longitudinal follow-up, limited understanding of how different clinical and demographic variables may have a mechanistic effect on study outcomes. Of clinical relevance, there was only one male participant represented across included studies, and therefore, future research should be inclusive of all genders including the LBGTQIA + patient populations. Future studies should consider embedding robust cost-effectiveness evaluation in all future prehabilitation studies.

## Supplementary Information

Below is the link to the electronic supplementary material.

Supplementary file1 (DOCX 27 kb)
